# Internet gaming disorder in an adolescent during the COVID-19 pandemic: a case report

**DOI:** 10.11604/pamj.2022.41.224.33941

**Published:** 2022-03-17

**Authors:** Novi Agung Rahmawati, Yunias Setiawati, Gusti Ayu Indah Ardani, Ekachaeryanti Zain, Victor Pereira-Sanchez

**Affiliations:** 1 Department of Psychiatry, Faculty of Medicine, Universitas Airlangga, Surabaya, Indonesia,; 2 Public Health Office, Kota Kediri, Jawa Timur, Indonesia,; 3 Division of Child and Adolescence, Department of Psychiatry, Faculty of Medicine, Universitas Airlangga, Surabaya, Indonesia; 4 Division of Child and Adolescence, Department of Psychiatry, Faculty of Medicine, Universitas Udayana, Bali, Indonesia,; 5 Department of Psychiatry, Faculty of Medicine, Mulawarman University, Samarinda, Indonesia,; 6 Department of Psychiatry, Columbia University, New York, United States,; 7 Division of Translational Epidemiology, New York State Psychiatric Institute, New York, United States,; 8 Department of Child and Adolescent Psychiatry, NYU Grossman School of Medicine, New York, United States,; 9 Department of Psychiatry, Amoud University, Borama, Somaliland, Somalia

**Keywords:** Internet gaming disorder, adolescent, behavioral addiction, COVID-19, case report

## Abstract

The internet has become an indispensable tool in people´s daily lives during the COVID-19 pandemic. Internet and video game use are experiencing rapid growth in the youth and adult populations as a major source of entertainment. However, excessive gaming may cause addiction and negatively impact mental health, entailing low psychosocial well-being, poor social skills, and decreased academic achievement. We report the case of a 16-year-old student with a “typical” pattern of internet gaming disorder (IGD) developed during the pandemic, which improved after weeks of treatment with pharmacotherapy and psychosocial interventions. This case highlights that it is essential for the mental health professionals to know the psychopathology of IGD and multimodal approaches to treat it.

## Introduction

The world is grappling with the COVID-19 pandemic [1]. Due to physical distancing policies, many people relies on the internet for daily routine activities [1-3]. Internet addiction has been conceptualized as a deficient ability to control internet use, leading to problems [4,5]. Internet use and video gaming are experiencing rapid growth among youth and adult populations, and internet gaming is one of the major sources of entertainment for people of all ages. Excessive gaming may negatively impact mental health and constitute behavioral addiction. Challenges in the diagnosis of internet gaming disorder (IGD) are due to its novelty and lack of broad and strong consensus; gaming disorder is still considered a “condition for further study” in the 5^th^ edition of the Diagnostic and Statistical Manual of Mental Disorders-5 (DSM-5) and has very recently been included as a behavioral addiction in the 11^th^ edition of the International Classification of Diseases (ICD-11) [6]. Gaming addiction is associated with a variety of comorbid psychiatric disorders including social phobia, generalized anxiety disorder, attention deficit hyperactivity disorder, panic disorder, depression, obsessive-compulsive disorder (OCD), and various psychosomatic symptoms. A recent research shows that a wide range of health and social problems are associated with excessive use of video games such as obesity, violence, anxiety, lower school performance, social phobia, and depression. In addition, certain risk factors for becoming pathological gamers have been identified, including poor baseline social competence, neuroticism, aggression and hostility, avoidant and schizoid interpersonal tendencies, greater impulsivity, loneliness, social inhibition, boredom tendencies, reduced self-control, and low self-esteem [2,6-8]. Clinically significant impairments are determined by the impact on daily life functioning which manifests as severe social, emotional, and occupational problems, including stress related to games rather than to real life, low psychosocial well-being, loneliness, poor social skills, decreased academic achievement, and aggressive/oppositional behavior [2,9]. Many of these behaviors are associated with comorbid OCD, being potentially susceptible to improve with selective serotonin reuptake inhibitors (SSRIs) and augmentation strategies [10].

## Patient and observation

**Patient information**: a 16-year-old male student was brought by his parents to the Child and Adolescent Psychiatry Outpatient Clinic at a general hospital. His mother said that he used to be a good student with high academic performance, and his parents had high expectations. At the beginning of the COVID-19 pandemic he used to play two hours after online school, but with time he started spending several hours a day on his phone and started playing video games during online school activities. He claimed that the internet was “the most important thing” in his life and that he gained pleasure when he used it. He also reported feeling a “compulsion” to game as an evasion of pandemic-related stress, including online education. His internet and gaming use increased day by day, and his academic performance declined. His mother complained about his behavior leading to decreased academic performance, poor sleep due to staying up late to play, decreased personal hygiene, irregular eating habits, neglect of important responsibilities, and aggression toward family members when they tried to prevent him from accessing video games: he would insult his parents, shout at them, and destroy household property. By that time he had no friends in real life, but he had made many virtual ones through online chat. He started suffering a lack of self-confidence and inferiority complex when dealing with his peers and teachers, as well as impaired social interaction with relatives. He progressively started to seem aloof and socially detached, and he abandoned school. He had no past or family history of psychiatric illness.

**Diagnostic assessment**: clinical assessments included a semi-structured interview collecting demographic information, the nature of its internet use, including time spent on “essential” (required for school) versus “nonessential” (pleasure, recreational, or personal) use. At the moment of initial assessment, he was reported to spend about 70 hours per week in “nonessential” and 15 hours in ‘essential´ use. His “nonessential” use comprised chat forums (10%), strategy games (50%), music (10%), and ‘surfing´ the web (30%). The assessment also included the Internet Addiction Diagnostic Questionnaire (KDAI). Patient´s score of KDAI was 249, meaning a positive screening test for internet addiction [1].

**Therapeutic intervention**: the patient and his parents refused hospitalization. Therefore, the patient was treated at home with regular psychiatric outpatient visits. Pharmacological treatment with fluoxetine was started (10 mg per day), with an increase to 20 mg per day within a month. At a follow-up visit after four weeks he reported “a little improvement”, with a reduction of internet use to about 50 hours per week in “nonessential” use, maintaining 15 hours in “essential” use. Pharmacological augmentation with quetiapine 50 mg/day was started, and the patient and family were provided psychoeducation to understand the negative consequences of IGD. Behavioral therapy sessions provided them with training in time management and encouraged them to replace online activities with healthy alternatives [10]. Initially, the patient rejected the indications to completely stop using video games, and therapy strategies of open-ended questioning, reflective listening, validating, and summarizing were employed, helping the patient to express his concerns about the changes. Two months later, he reported spending about 15 hours per week in “nonessential” and 20 in “essential” use and noted that he was able to exert control. This improvement was maintained during the 4-month follow-up by using the same medication.

**Informed consent**: the patient and his mother signed the written consent for the publication of this case report. Some specific details without clinical relevance may have been changed to protect patient´s anonymity.

## Discussion

This case reports a “typical” problematic internet use with the following features: (a) uncontrollable, (b) markedly distressing, time-consuming, or resulting in social and occupational (school performance) issues, (c) not solely present during hypomanic or manic symptoms. This case report illustrates the existence of behaviors similar to those of subjects with substance and gambling addictions as defined in the DSM-5. Its manifestations include withdrawal, tolerance, as well as work and social impairment. Under DSM-5, IGD refers to the “persistent and recurrent use of the internet to engage in games, often with other players, leading to clinically significant impairment or distress as indicated by five (or more) of the nine diagnostic criteria (preoccupation or obsession, withdrawal, tolerance, loss of control, loss of interest, continued overuse, deceiving, escape of negative feelings, functional impairment) in 12 months” [7,11-14]. Criteria for problematic internet use: (a) too preoccupied with the internet, such as: 1) always wanting to use the internet (unbearable), 2) using the internet more than planned; b) internet addiction that causes clinical impairment or impairment in social, occupational, or other important functions; c) there is no excessive internet use exclusively during periods of hypomania or mania [6]. The patient involved in this study meets all of them. Withdrawal is the unpleasant feeling or physical effects that occur when the problematic substance or behavior is not available or removed; the patient involved in this study became irritable when unable to play games. Tolerance refers to increasing amounts of a substance or behavior needed to achieve the previous effects [14]. In addition, a self-reported tool to assess psychopathology of internet addiction developed and validated in Indonesia, the Internet Addiction Diagnostic Questionnaire (KDAI), was used to assess the patient. The KDAI has good reliability and validity as screening tool for internet addiction in adolescents. It consists of 7 domains and 44 statement items with good content and construct validity [15].

The patient involved in this study progressively increased his gaming time to obtain euphoria, escape from stress, and feel rewarded through his game. A study showed that internet addiction sufferers are significantly more sensitive to rewards than controls and they appear to be less sensitive to the negative consequences of their internet engagement. A person is more likely to engage in gaming behavior again when feeling rewarded. When the reward becomes unpredictable but frequent enough (intermittent reward), it can exert the strongest effect on increasing the behavior [16]. Additionally, the patient continued to game and neglected his school responsibilities, and became socially withdrawn [15,16].To note, the problems of this patient emerged during the COVID-19 pandemic, a global context that has forced many people to use the internet almost for all activities in their life such as working, studying, interacting socially, getting entertainment, shopping, etc., and making people more vulnerable to develop or worsen problematic use of the internet [17]. The patient involved in this case report used the internet for school during the beginning of the COVID-19 pandemic. However, the prolonged exposure to the internet, feelings of boredom and psychosocial stress eventually made him increase his “non-essential” screen time to the level of a clinical disorder.

A case of severe internet gaming disorder recently reported in Spain showed a patient that required hospital treatment for “detoxification” [18]. In our case, the patient and his parents refused the hospitalization, and outpatient pharmacotherapy and psychological interventions for the patient and his family were effective. Individuals with IGD tend to display obsessive-like characteristics related to their computer/internet use. However, though OCD tends to respond well to SSRIs, the study patient did not respond enough to this pharmacotherapy alone within the observed duration of treatment. One limitation of this case report is that fluoxetine might not have been given for long enough or at a high enough dose to obtain optimal effect before quetiapine was added. Psychotherapy and psychoeducational approaches can be used as the main treatment for addiction, despite the use of additional drugs [6,10,19]. Antidepressants and antipsychotics have both been used with varying degrees of success, along with other pharmacologic agents. The clinical experience reported in scientific literature has shown that SSRIs (i.e., fluoxetine, citalopram, clomipramine, fluvoxamine, sertraline, escitalopram) and antipsychotic medications (i.e., quetiapine) have been used to treat internet addiction and IGD. SSRI (in the case reported, fluoxetine) may suppress inhibitory responses and control compulsive repetition, which likely explains its effectiveness in treating IGD. It has been hypothesized that atypical antipsychotics (in the case reported, quetiapine) might be a safe and effective augmenting medication to remediate address behavioral issues associated with impulsivity, including IGD [10].

Family therapy should focus on several key areas: (a) educate parents about the negative consequences of IGD, (b) stop blaming the patient for addictive behaviors, (c) improve open communication about premorbid problems in the family in search of psychological fulfillment (d) encourage parents to help addiction recovery through listening to patient´s feelings, finding new hobbies, or taking a long vacation. Family support may help the patient to recover from IGD. Education about the negative consequences of IGD and parental monitoring is an important protective factor for internet addiction, especially for high school students. Parents play a major role in helping their children by setting goals and building confidence, addressing addictive behaviors, and learning new behavioral skills. Being a supportive parent includes considering children's interests, having reasonable expectations, and providing encouragement, reinforcement, and some insight into problem-solving [7,20-23].

## Conclusion

Mental health professionals should be familiar with the psychopathology of IGD and internet addiction, and its consequential problems such as disrupted conduct, social withdrawal, aggression, and poor school performance. Clinical experience reported in the literature and this case suggest that the combination of pharmacological treatment such as antidepressants (SSRI) and antipsychotics with individual and family-focused psychosocial interventions are useful in improving problematic behaviors.
